# Serum uric acid/creatinine ratio and osteoporosis in the elderly: a NHANES study

**DOI:** 10.3389/fmed.2025.1530116

**Published:** 2025-04-25

**Authors:** Jiaying Yu, Chuyun Xu, Dongchi Ma, Yu Li, Lili Yang

**Affiliations:** School of Nursing, Zhejiang Chinese Medical University, Hangzhou, Zhejiang, China

**Keywords:** osteoporosis, elderly, serum uric acid, creatinine, NHANES

## Abstract

**Background:**

Osteoporosis (OP) is a metabolic bone disorder that is of significant concern to the elderly. However, few studies have investigated the correlation between the serum uric acid to creatinine ratio (UA/Cr) and OP in elderly individuals. This research seeks to examine the connection between UA/Cr levels and OP in older adults.

**Methods:**

Data on participant information for the study was obtained from four cycles of the NHANES database. Multivariable logistic regression was employed to examine the correlation between UA/Cr and OP, adjusting for potential confounders such as age, gender, and race. The diagnostic efficacy of UA/Cr for OP was evaluated utilizing ROC curves.

**Results:**

Multivariable logistic regression analysis showed that serum UA/Cr levels were significantly lower in individuals with OP than in those without OP. (OR = 0.83 [0.76, 0.91], *P* < 0.001). Subgroup analyses indicated a stronger association in men (OR = 0.77 [0.64, 0.94], *P* = 0.009) and women (OR = 0.85 [0.76, 0.95], *P* < 0.003). Furthermore, multivariable logistic regression analyses by ethnicity revealed that this association was significant solely among Non-Hispanic Whites (OR = 0.78 [0.68, 0.90], *P* < 0.001). The area under the ROC curve (AUC) for UA/Cr in predicting OP was higher than that for SUA alone, indicating superior predictive value.

**Conclusion:**

A higher UA/Cr level within the normal range is associated with a lower risk of OP, providing insights for its diagnosis and risk assessment.

## 1 Introduction

Osteoporosis (OP) is a chronic metabolic bone disease characterized by reduced bone mass and deterioration of bone microstructure. This condition results in a significant reduction in bone strength and an elevated susceptibility to fractures, especially among the aged population (1). OP is often asymptomatic in its early stages, with minimal discomfort. As the disease advances, patients may experience various clinical manifestations, including low back pain or generalized bone pain. In severe instances, fractures may arise from minor activities or falls, leading to kyphosis, enforced prolonged bed rest, and infections, significantly impairing patients’ quality of life (2). Osteoporotic fractures impact up to 50% of females and 20% of males over 50 (3). In 2017, the worldwide population aged 60 years and over was 962 million, and it is projected that this demographic would increase to 2.1 billion by 2050, approximately 20% of the total global population (4). Due to the rapid aging of the world population and rising life expectancy, osteoporosis will gradually emerge as a global epidemic, inflicting a significant economic burden on nations and society. In the United States, approximately 2 million osteoporotic fractures occur annually, a figure projected to exceed 3 million fractures per year by 2040. The associated costs exceed $95 billion per year, with comparable expenses observed in other developed countries (5, 6). It is estimated that more than 23 million people in the European Union are at high risk of osteoporotic fractures, with nearly one-quarter of annual deaths attributable to hip or spine fractures. Osteoporosis and the fragility fracture it causes result in losses exceeding €56 billion annually to the European healthcare system (7). Consequently, early identification and intervention are essential for seniors regarding the prognosis of osteoporosis and mitigating the related healthcare burden. Uric acid (UA), a byproduct of purine metabolism, is increasingly recognized via experimental and clinical studies for its significant significance as an antioxidant and its protective function in bone metabolic diseases (8). Research has shown that higher serum uric acid (SUA) levels are associated with increased bone mineral density (BMD), lower bone turnover, and a reduced risk of vertebral fractures, suggesting that uric acid may have a protective role in bone metabolism (9). It is thought to exert this effect by neutralizing free radicals, thereby protecting bone tissue from oxidative stress. Due to its antioxidant properties, UA may also inhibit osteoclast-mediated bone resorption and help maintain higher BMD (10). However, uric acid can also promote oxidative stress through superoxide radicals generated by NADPH oxidase. The imbalance between oxidative stress and antioxidation can affect bone remodeling and lead to osteoporosis (11). Elevated uric acid levels may also result in gouty arthritis, triggering chronic inflammation, which in turn stimulates osteoclast activity and increases bone resorption (12). Some studies suggest a non-linear, inverted U-shaped relationship between 25(OH)D and SUA (13, 14), indicating that the relationship between uric acid levels and bone health may follow a ‘U’ shaped curve, where both low and high uric acid levels could have detrimental effects on bone metabolism (15). Nevertheless, serum uric acid (SUA) is significantly affected by renal function, and elevated SUA has been identified as a biomarker of impaired renal function. Because of the impact on renal function, the SUA indicator alone can no longer accurately represent the actual SUA level in the body. The SUA indication alone can no longer accurately represent the genuine SUA level in the body due to the impact of renal function. Creatinine (Cr) serves as a biological marker of renal function, whereas the serum uric acid/creatinine (UA/Cr) ratio is an emerging biomarker that mitigates the influence of renal excretion on SUA, thus providing a standardized indicator of SUA that is thought to represent endogenous UA levels (16). The relationship between UA/Cr and OP remains underexplored in existing research. While UA/Cr is known to be linked to several chronic metabolic disease (17, 18), its association with OP has not been extensively studied. The diagnosis of osteoporosis mostly depends on imaging tests, and investigating serological markers that help in the early detection of osteoporosis is highly significant for its diagnosis and treatment. This study utilized a representative certified sample from the National Health and Nutrition Examination Survey (NHANES) to explore the correlation between UA/Cr and OP in people aged ≥60 years. The study evaluated the predictive value of UA/Cr for OP and compared the differences between UA/Cr and SUA in predicting OP.

## 2 Materials and methods

### 2.1 Research design and population

This study employed a cross-sectional, population-based research methodology to assess secondary data. The research design is cross-sectional in nature, as it involves the analysis of data collected at a single point in time from the target population. This study utilized data from the NHANES database, a national survey representing the U.S. population. It employs a complex, multi-stage probability sampling method to gather comprehensive data on the dietary habits and overall health of the general U.S. population. This research integrated NHANES data from the years 2007–2008, 2009–2010, 2013–2014, and 2017–2018. Study participants were adults aged 60 and older who completed the NHANES interview and assessment. Participants with malignant tumors, thyroid diseases, and those lacking complete data on blood UA, creatinine, and femoral bone mineral density (BMD) were eliminated from the study.

### 2.2 Study variables

#### 2.2.1 Measurement of SUA and Cr

The exposure variables in this study were SUA and Cr. The Beckman Synchron LX20 biochemical analyzer was utilized in 2007, while the Beckman Coulter UniCel^®^ DxC800 automatic biochemical analyzer was employed post-2008. SUA is identified by a timed endpoint experiment. The concentration of Cr was determined by the Jaffe rate method (alkaline picric acid kinetics), and the observed absorbance was a direct measure of the creatinine concentration in the sample. The assay was changed with the Roche/Hitachi Cobas 6000 analyzer after 2017, and the detection methods have also changed. The hydrogen peroxide generated from the oxidation of SUA by uricase was catalyzed by peroxidase in the presence of 4-aminobenzazole, resulting in a quantifiable colored product. Concurrently, the determination of Cr was substituted with an enzymatic approach.

#### 2.2.2 BMD measurements and definition of OP

BMD measurements were conducted with a Hologic QDR - 4500A fan beam densitometer model dual-energy x-ray bone densitometer. OP is characterized as any femoral region exhibiting a BMD score that is under −2.5 deviations from average compared to the baseline population of younger individuals (19). In this study, the evaluation included the entire femur as well as specific regions such as the femoral neck, trochanter, and intertrochanter. The OP diagnostic thresholds for these areas were found to be 0.68 g/cm^2^ for the whole femur, 0.59 g/cm^2^ for the femoral neck, 0.49 g/cm^2^ for the trochanter, and 0.78 g/cm^2^ for the intertrochanter (20).

#### 2.2.3 Covariates

In NHANES, data were gathered by a standardized participant questionnaire, and a medical assessment was performed for each participant. The study examined several covariates, including age, sex, race, educational attainment, the poverty-to-income ratio (PIR), times of moderate physical activity, smoking and drinking status, as well as biochemical markers such as blood urea nitrogen (BUN), calcium, phosphorus, total cholesterol, and total protein. The moderate activity status was derived from the inquiry regarding “Minutes of moderate recreational activities.” The drinking status was ascertained from the inquiry, “4/5 or more drinks daily.” In this study, several known confounding variables were selected to control for potential factors influencing the development of osteoporosis, ensuring the reliability of the results. Age is a well-established risk factor for osteoporosis, as bone density typically decreases with age. Gender differences also play a significant role, particularly in postmenopausal women, who are at a higher risk (21). Racial variations in bone density susceptibility necessitate its inclusion as a confounder to ensure generalizability (22). Education level, reflecting socio-economic status, impacts health behaviors and access to medical resources, making it an essential covariate. The PIR serves as a proxy for socio-economic status, influencing lifestyle and health resources. Physical activity is crucial for bone health, and its duration was controlled to reduce confounding (23). Smoking and excessive alcohol consumption are well-known risk factors, affecting bone metabolism and formation, thus requiring adjustment (24). Biochemical markers such as BUN, calcium, phosphorus, total cholesterol, and total protein are directly related to bone metabolism and nutritional status. These markers were controlled to minimize their potential confounding effects. By incorporating these variables, the study aimed to account for established osteoporosis risk factors, thereby reducing bias and enhancing the accuracy and validity of the findings.

### 2.3 Statistical analysis

Data were analyzed utilizing R^1^ and EmpowerStats software packages, with *P* < 0.05 being statistically significant. The continuous statistics were reported as mean ± standard error (SE), whereas categorical variables as percentages. Continuous and categorical variables were analyzed between groups utilizing the weighted linear regression and weighted chi-square test, respectively. Linear regression was employed to assess the relationship between continuous variables across different groups, as it is suitable for evaluating how continuous predictors influence a dependent variable. The association between UA/Cr and OP was assessed through multivariate logistic regression models, which are appropriate for analyzing the relationship between multiple independent variables and a categorical dependent variable with two or more categories. We chose multivariate logistic regression because the outcome variable in our study is the presence or absence of osteoporosis, a categorical variable, and distinct analyses performed for different genders and ethnic groups. To explore the nonlinear relationship between UA/Cr and OP, smoothed curve fitting and generalized summation models were utilized. Finally, receiver operating characteristic (ROC) curves were employed to compare the predictive accuracy of UA/Cr versus SUA for diagnosing OP.

## 3 Results

### 3.1 Selection of study population

**FIGURE 1:**
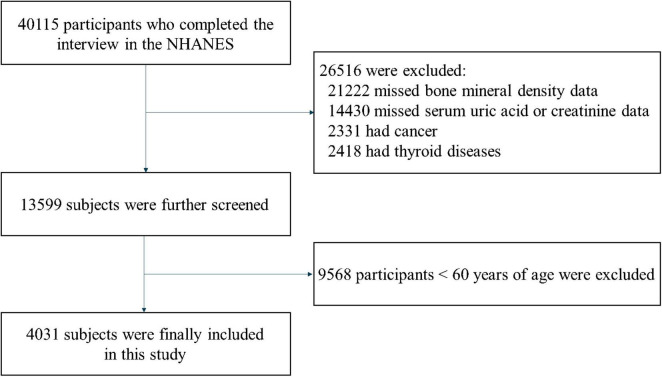
Study population selection flowchart.

Utilizing data from four cycles of NHANES (2007–2008, 2009–2010, 2013–2014, and 2017–2018), and excluding individuals with absent SUA or Cr data (*n* = 14,430), those lacking femoral BMD data (*n* = 21,222), individuals with thyroid disorders (*n* = 2,418), and cancer patients (*n* = 2,331), a combined total of 4,031 those involved aged 60 years or above were enrolled (Figure 1).

### 3.2 Clinical features of the participants in the study

Table 1 displays the weighted attributes of the study subjects. The mean age was 68.50 years. The gender distribution comprised 47.75% women and 52.25% men, with the predominant demographic being Non-Hispanic Whites at 73.22%. The quartiles of UA/Cr are as follows: Q1: 0.28–3.40; Q2: 3.41–4.06; Q3: 4.07–4.81; Q4: 4.82–9.92. Significant disparities in baseline parameters were seen among UA/Cr quartiles for sex, age, race, BUN, blood phosphorus, total protein, total cholesterol, and OP. Participants exhibiting higher UA/Cr ratios were predominantly Non-Hispanic White and Non-Hispanic Black, characterized by a younger age, lower blood phosphorus and BUN levels, and higher protein and total cholesterol levels. Furthermore, people in the elevated UA/Cr quartile exhibited a lower likelihood of osteoporosis. The observed differences in blood phosphorus, BUN, and protein levels across quartiles may reflect renal handling of UA and phosphate homeostasis, potentially linking UA/Cr ratios to bone metabolism pathways. Lower BUN and higher protein levels in high UA/Cr groups suggest improved nutritional status, which could partially explain their reduced osteoporosis risk.

**TABLE 1:** Weighted attributes of the study cohort based on UA/Cr.

	Total	Q1	Q2	Q3	Q4	*P*-value
		**(0.28–3.40)**	**(3.41–4.06)**	**(4.07–4.81)**	**(4.82–9.92)**	
**Sex (%)**						<0.0001
Male	52.25	57.92	53.93	55.30	41.13	
Female	47.75	42.08	46.07	44.70	58.87	
**Age (years)**	68.50 (0.14)	69.87 (0.23)	68.84 (0.30)	67.90 (0.30)	67.27 (0.23)	<0.0001
**Race/ethnicity (%)**						<0.0001
Mexican American	5.42	4.09	5.24	6.06	6.38	
Other Hispanic	4.54	3.37	4.26	5.11	5.53	
Non-Hispanic White	73.22	76.82	75.33	72.42	67.91	
Non-Hispanic Black	9.68	12.07	9.97	7.97	8.57	
Other race/ethnicity	7.13	3.66	5.20	8.44	11.61	
**Level of education (%)**						0.0797
Less than high school	20.18	20.16	18.20	18.79	23.77	
High school	26.22	26.52	25.14	26.00	27.27	
More than high school	53.45	53.21	56.61	54.83	48.88	
Not reported	0.15	0.10	0.06	0.38	0.08	
**PIR**	3.03 (0.05)	2.96 (0.08)	3.03 (0.09)	3.14 (0.09)	2.98 (0.09)	0.4187
**Moderate activities (minutes)**	65.33 (2.41)	62.77 (5.11)	73.39 (5.52)	62.19 (3.43)	62.09 (4.10)	0.2319
**Smoked at least 100 cigarettes in life (%)**						0.5876
Yes	50.72	51.26	51.70	48.09	51.84	
No	49.23	48.74	48.16	51.87	48.16	
Not reported	0.05	0.00	0.15	0.04	0.00	
**Alcohol consumption**						0.0917
Nondrinker or Moderate alcohol use	14.94	15.43	15.48	13.23	15.61	
High alcohol use	68.50	68.80	71.47	67.58	65.96	
Not reported	16.56	15.77	13.05	19.19	18.43	
**BUN (mmol/L)**	5.90 (0.05)	6.74 (0.10)	5.91 (0.07)	5.59 (0.09)	5.31 (0.10)	<0.0001
**Serum total calcium (mmol/L)**	2.36 (0.00)	2.35 (0.00)	2.36 (0.00)	2.36 (0.00)	2.36 (0.00)	0.3686
**Serum phosphorus (mmol/L)**	1.19 (0.00)	1.21 (0.01)	1.20 (0.01)	1.16 (0.01)	1.19 (0.01)	0.0075
**Total protein (g/L)**	70.13 (0.15)	69.46 (0.22)	69.64 (0.21)	70.20 (0.21)	71.30 (0.21)	<0.0001
**Total cholesterol (mmol/L)**	5.00 (0.03)	4.81 (0.05)	4.97 (0.04)	5.12 (0.04)	5.10 (0.06)	<0.0001
**OP (%)**						0.0415
Yes	86.69	15.31	15.24	10.99	11.49	
No	13.31	84.69	84.76	89.01	88.51	

Mean (SE) for continuous variables: the *P*-value was computed using the weighted linear regression model. (%) for categorical variables: the *P*-value was computed using the weighted chi-square test.

### 3.3 Relationship between UA/Cr and OP

**FIGURE 2:**
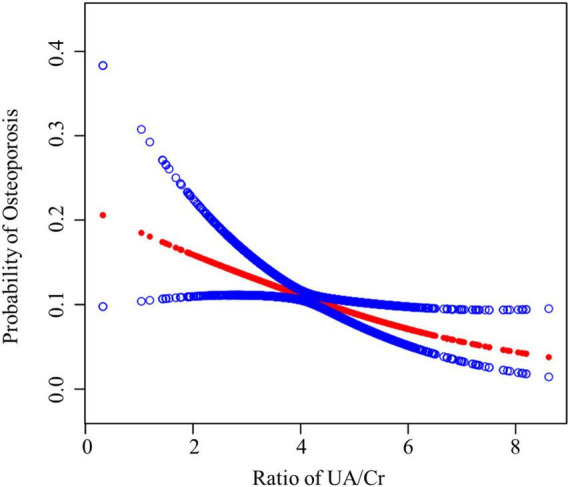
Relationship between UA/Cr and OP. The red line represents the smooth fitting curve between the variables. The blue line represents the 95% confidence interval for the goodness of fit. Age, sex, race/ethnicity, education, PIR, moderate activities, smoking behavior, alcohol consumption, BUN, serum total calcium, serum phosphorus, total protein, and total cholesterol were adjusted.

Table 2 presents the results of the multiple regression analysis. The correlation between UA/Cr levels and the incidence of OP was demonstrated by both continuous and categorical analysis. Ongoing analyses indicated a significant negative correlation between UA/Cr levels and the occurrence of OP (OR = 0.83 [0.76, 0.91], *P* < 0.001). Categorical analyses indicated that, using the lowest UA/Cr level (0.28–3.40) as a reference, higher UA/Cr levels (4.82–9.92) remained significantly associated with the prevalence of OP after adjusting for covariates (Model 1: OR = 0.80 [0.62, 1.03], *P* = 0.088; Model 2: OR = 0.75 [0.57, 0.99], *P* = 0.041; Model 3: OR = 0.64 [0.48, 0.87], *P* = 0.004). Individuals in the highest quartile of UA/Cr exhibited a lower risk of OP compared to those in the lowest quartile (OR = 0.64 [0.48, 0.87], *P* < 0.001). This dose-response relationship (progressive OR reduction from Q1 to Q4) supports a potential biological gradient, consistent with uric acid’s proposed antioxidant effects in bone remodeling. Figure 2 illustrates the smoothed curve fitting and generalized summation model that depicts the correlation between UA/Cr and the prevalence of OP.

**TABLE 2:** Association between UA/Cr ratio and OP in the multiple regression model.

Variable	Model 1		Model 2		Model 3	
	**OR (95% CI)**	***P*-value**	**OR (95% CI)**	***P*-value**	**OR (95% CI)**	***P*-value**
**UA/Cr ratio**	0.90 (0.83, 0.97)	0.009	0.88 (0.80, 0.96)	0.003	0.83 (0.76, 0.91)	<0.001
**UA/Cr categories**						
Q1(0.28–3.40)	Reference		Reference		Reference	
Q2(3.41–4.06)	1.01 (0.79, 1.29)	0.943	1.01 (0.78, 1.32)	0.934	1.02 (0.77, 1.33)	0.910
Q3(4.07–4.81)	0.73 (0.57, 0.95)	0.021	0.75 (0.57, 0.99)	0.046	0.68 (0.51, 0.91)	0.010
Q4(4.82–9.92)	0.80 (0.62, 1.03)	0.088	0.75 (0.57, 0.99)	0.041	0.64 (0.48, 0.87)	0.004
P for trend		0.017		0.010		<0.001
**Sex categories**						
Male	0.70 (0.59, 0.83)	<0.001	0.81 (0.68, 0.97)	0.020	0.77 (0.64, 0.94)	0.009
Female	0.84 (0.76, 0.92)	<0.001	0.90 (0.82, 0.99)	0.039	0.85 (0.76, 0.95)	0.003
**Race/ethnicity categories**						
Mexican American	0.94 (0.76, 1.17)	0.590	0.92 (0.72, 1.16)	0.467	0.91 (0.70, 1.20)	0.508
Other Hispanic	0.84 (0.65, 1.08)	0.178	0.80 (0.60, 1.05)	0.107	0.78 (0.58, 1.05)	0.098
Non-Hispanic White	0.80 (0.70, 0.90)	<0.001	0.79 (0.69, 0.90)	<0.001	0.78 (0.68, 0.90)	<0.001
Non-Hispanic Black	0.90 (0.72, 1.14)	0.388	0.86 (0.67, 1.09)	0.217	0.83 (0.63, 1.09)	0.182
Other race/ethnicity	1.08 (0.86, 1.34)	0.513	0.93 (0.73, 1.18)	0.566	0.94 (0.73, 1.20)	0.609

CI, confidence interval. OR, odds ratio. Model 1: no covariates were adjusted. Model 2: age and sex were adjusted. Model 3: age, sex, race/ethnicity, education, PIR, moderate activities, smoking behavior, alcohol consumption, BUN, serum total calcium, serum phosphorus, total protein, and total cholesterol were adjusted.

In the analysis conducted on subgroups categorized by gender and ethnic background, the inverse association between UA/Cr levels and the prevalence of OP remained significant in males (OR = 0.77 [0.64, 0.94], *P* = 0.009) and females (OR = 0.85 [0.76, 0.95], *P* < 0.003). Additionally, among Non-Hispanic Whites, the association was also significant (OR = 0.78 [0.68, 0.90], *P* < 0.001). Nonetheless, it was not statistically significant among Mexican Americans (OR = 0.91 [0.70, 1.20], *P* = 0.508), other Hispanics (OR = 0.78 [0.58, 1.05], *P* = 0.098), Non-Hispanic Blacks (OR = 0.83 [0.63, 1.09], *P* = 0.182), and those of other races/nationalities (OR = 0.94 [0.73, 1.20], *P* = 0.609). Subgroup analyses demonstrated ethnic-specific associations, with a significant inverse correlation observed exclusively in Non-Hispanic Whites (OR = 0.78, *P* < 0.001), whereas no statistically significant associations were detected in other ethnic groups. This disparity may be attributable to multiple factors: (1) Genetic variations in urate transporter genes show ethnic specificity, with differential allele frequencies across populations affecting urate homeostasis. Notably, specific SLC2A9 variants, which are more frequent in Non-Hispanic Whites, significantly elevate blood uric acid levels through enhanced uric acid reabsorption (25); (2) Pathophysiological heterogeneity in osteoporosis, including racial differences in vitamin D metabolism, as evidenced by the notably lower prevalence of vitamin D deficiency in Non-Hispanic Whites compared to other racial groups (26); and (3) Potential limitations in statistical power for smaller subgroup analyses. Further studies are warranted to validate these associations and investigate potential gene-environment interactions underlying these ethnically divergent patterns.

### 3.4 ROC curves to assess the predictive value of UA/Cr and SUA for OP

**FIGURE 3:**
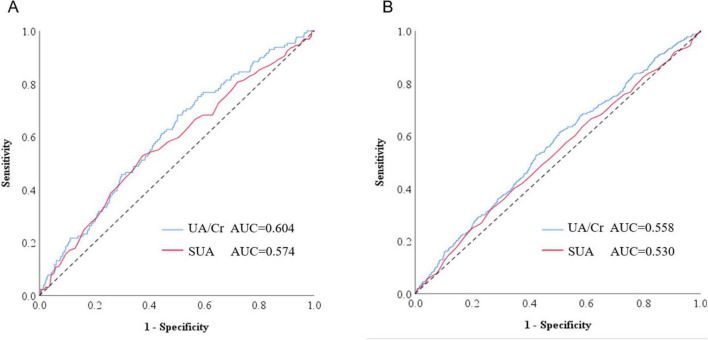
The ROC curve of UA/Cr and SUA with osteoporosis. **(A)** The prediction of UA/Cr and SUA for osteoporosis in men. **(B)** The prediction of UA/Cr and SUA for osteoporosis in women.

The ROC curve was employed to evaluate and compare the diagnostic efficacy of UA/Cr and SUA in identifying OP. UA/Cr and SUA served as detection variables, whereas OP functioned as the outcome variable, stratified by gender. Figure 3 illustrates that the area under the curve (AUC) for UA/Cr in evaluating the incidence of OP in men was 0.604 (95% CI: 0.555–0.653, *P* < 0.001), surpassing the AUC for SUA in assessing the occurrence of OP, which was 0.574 (95% CI: 0.521–0.626, *P* = 0.005) (Figure 3A). The AUC for UA/Cr to assess the incidence of OP in women was 0.558 (95% CI: 0.527–0.590, *P* < 0.001), while the AUC for SUA in assessing the occurrence of OP was 0.530 (95% CI: 0.497–0.562, *P* = 0.070) and was not statistically significant. Though modest, UA/Cr’s superior discriminative capacity over SUA suggests clinical utility as a complementary biomarker. The gender disparity in predictive performance (male AUC = 0.604; female AUC = 0.558) may reflect testosterone’s dual role in both uric acid production and bone protection. Notably, SUA’s non-significant association with OP in women (*P* = 0.070) underscores the importance of creatinine normalization, which may mitigate confounding by muscle mass differences — a critical consideration in aging populations.

## 4 Discussion

As the population ages, osteoporosis has emerged as a prevalent societal concern globally. OP is the predominant form of metabolic bone disease and the leading cause of fractures in the elderly (27). If not addressed promptly, it may lead to a range of consequences, including bodily pain and fractures, significantly diminishing quality of life. The complications caused by OP have brought a heavy economic burden to every country, and the related costs in the United States are expected to exceed $ 95.9 billion by 2040 (28). Therefore, the early identification of diminished bone mineral density and subsequent intervention is crucial for the prevention and management of osteoporosis. This study utilized a large, representative sample from the American population to investigate the relationship between UA/Cr levels and the incidence of OP in the elderly. Multivariate logistic regression analysis showed that there was a negative correlation between the two, which was similar to the results of Lin et al. (29).

Particularly, the dual role of UA in bone metabolism requires careful interpretation. Within physiological ranges (3–6 mg/dL), UA exhibits potent antioxidant properties by scavenging reactive oxygen species (ROS) and inhibiting NADPH oxidase activity, thereby protecting osteoblasts from oxidative damage (30). This aligns with our findings where moderate UA/Cr ratios correlated with reduced OP risk. Conversely, hyperuricemia (>7 mg/dL) triggers pro-oxidative effects through xanthine oxidase activation, generating superoxide anions that promote osteoclast differentiation via RANKL/OPG pathway dysregulation (31). This J-shaped relationship may explain contradictory findings in prior studies. SUA is the ultimate byproduct of purine metabolism in the human body. Recent researches have verified that UA possesses antioxidant properties within a specific range (32). Inflammatory cytokines and oxidative stress are implicated in the pathophysiological mechanisms of osteoporosis. Specifically, UA’s antioxidant capacity suppresses NF-κB signaling in osteoblasts, reducing IL-6 and TNF-α production, which are known to accelerate bone resorption (33). However, excessive UA induces NLRP3 inflammasome activation in macrophages, amplifying IL-1β secretion and osteoclastogenesis (34). Oxidative stress is regarded as a significant contributor to bone loss and the degradation of bone microstructure. Oxidative stress can stimulate osteoclast activation and enhance bone resorption, while concurrently impairing osteoblast function and influencing bone formation. The antioxidant capabilities of UA may mitigate these processes and hence provide a preventive function in osteoporosis. Emerging evidence from cognitive health research suggests that tailored exercise programs, such as aerobic and resistance training, may further alleviate oxidative stress by enhancing neurotrophic factors and improving systemic antioxidant capacity, thereby offering dual protection against cognitive decline and bone metabolism dysregulation (35). These findings, although primarily focused on cognitive outcomes, highlight a potential cross-domain protective role of exercise in mitigating oxidative stress-related pathologies, including bone metabolism dysregulation.

The antioxidant properties of SUA have attracted significant scholarly interest regarding its association with OP. Xiang Li et al. showed in a study of non-diabetic older adults that elderly people with OP had lower SUA levels than non-osteoporosis elderly people (36). Renwei Wang et al. identified a favorable correlation between SUA concentrations and lumbar BMD in American males (37). Xiaocong Yao et al. demonstrated a beneficial relationship between SUA and lumbar BMD in most older Americans; however, in the black population, this association demonstrates an inverse U-formed pattern (38). This racial disparity may stem from genetic polymorphisms in URAT1 (SLC22A12) affecting urate handling, as demonstrated by African ancestry-specific variants associated with 15% lower UA reabsorption (25). A study conducted in China to explore the correlation between SUA and BMD among postmenopausal women found that SUA concentrations did not significantly influence the risk of OP (39). In contrast to previous findings, Tanaka et al. identified increased SUA levels as a contributing factor to vertebral fractures in postmenopausal women who have type 2 diabetes (40). This discrepancy highlights the modifying effect of comorbidities—diabetes-induced advanced glycation end products (AGEs) may synergize with UA to exacerbate bone fragility through collagen crosslinking and osteocyte apoptosis (41). These variations highlight the need to consider population-specific factors, including ethnicity, comorbidities, and individual characteristics, when assessing the role of SUA in bone health. Therefore, clinical studies on the link between SUA and both BMD and OP are currently controversial. These disagreements may be caused by variations in the research design, the research samples, the ethnic makeup of the samples, and the controlled confounding variables.

Mechanistically, UA/Cr integrates two critical systems: purine metabolism and muscle-bone crosstalk. Cr, as a surrogate for muscle mass, reflects mechanical loading on bones—a key determinant of bone remodeling (42). The UA/Cr ratio thus encapsulates both biochemical (antioxidant) and biomechanical (muscle-derived anabolic stimuli) protective mechanisms. This dual representation may explain its superior predictive value over SUA alone, particularly in males who generally exhibit higher muscle mass (43). Cr is a primary metabolite of skeletal muscle, and its concentration is closely linked with skeletal muscle mass (44). Each unit of muscular tissue contains an equivalent amount of Cr as a unit of Cr, which is eliminated by the kidneys as urine. Consequently, human Cr levels can indicate muscle mass and renal function to some degree (45). This study employed UA/Cr as an exposure variable to mitigate the bias associated with utilizing SUA alone, as SUA is predominantly excreted via the kidneys, and compromised renal function may distort the actual UA levels in the body. In contrast, UA/Cr accounts for renal interference in SUA excretion and accurately represents SUA levels following standardized renal function, thereby more accurately reflecting the relationship between SUA and disease. Kawamoto et al. found that baseline UA/Cr was significantly associated with the frequency of metabolic syndrome among females in a community following a 3-year follow-up (46). A 6-month cohort study in Japan demonstrated that baseline UA/Cr ratio corresponded with an annual rate of decrease in glomerular filtration rate in diabetic patients after controlling for confounding variables, and it was an independent predictor of impaired renal function in patients with diabetes (47). An Italian national multicenter study (48) followed up 20724 subjects for 126 ± 64 months and found that UA/Cr was a separate risk indicator for cardiovascular events. However, there are relatively few studies investigating the link between UA/Cr and OP. This study suggests that the decrease of UA/Cr may be implicated in the occurrence of OP, which may be attached to the antioxidant properties of SUA (49). Furthermore, low UA/Cr may signal concurrent muscle wasting (sarcopenia) and reduced antioxidant capacity—a “double-hit” mechanism for bone loss (50). Mitigate the creation of oxygen free radicals in osteoclast precursors by suppressing oxidative stress, so reducing bone formation and consequently decreasing the rate of bone turnover. Nonetheless, the precise mechanism remains ambiguous and requires validation through extensive clinical and fundamental research.

Despite its antioxidant properties, hyperuricemia (HUA) can lead to gout, hypertension, cardiovascular disease, and other health complications (51, 52). HUA can be defined as SUA levels over 6.0 mg/dL (360 μmol/L) in females and 7.0 mg/dL (420 μmol/L) in males (53). Increased SUA levels can result in the accumulation of urate crystals in the joints, kidneys, and other locations, causing harm to these organs. Crystalline UA activates the NLRP3 inflammasome in macrophages, triggering IL-1β-mediated bone marrow stromal cell pyroptosis and impairing osteogenic differentiation (54). Additionally, soluble UA at pathological concentrations inhibits NO synthase in endothelial cells, reducing vascular supply to bone tissue and promoting hypoxic osteonecrosis (55). Research indicates that SUA levels in individuals with hyperuricemia are inversely connected with bone mineral density, thus raising the likelihood of osteoporosis. It may be related to the deposition of urate crystals in the kidney of patients with hyperuricemia, and the decrease of 1α-hydroxylase activity, resulting in a decrease in intestinal absorption of calcium and affecting bone metabolism (56, 57). Therefore, maintaining the high SUA level within the normal range may have positive significance for the prevention of OP, but at the same time, attention should be paid to controlling the SUA level to avoid the health hazards caused by hyperuricemia.

To explore the differences in this correlation across sex and race, further subgroup analyses were done in this study, which proved that higher UA/Cr levels were linked with a lower incidence of OP in both elderly male and female, and that this correlation was more pronounced in male. This sexual dimorphism may arise from testosterone’s role in upregulating muscle mass, creating a protective effect. Subgroup analysis categorized by race or ethnicity revealed that only the UA/Cr level in Non-Hispanic Whites exhibited a correlation with the incidence of OP, paralleling the findings of Vásquez et al. (58), while lifestyle factors, such as differences in exercise habits, may elucidate this discrepancy (59). Furthermore, specific variants of the SLC2A9 gene (such as rs7442295 and rs734553) are more prevalent in the Non-Hispanic White population, and these variants can significantly elevate serum uric acid levels by enhancing urate reabsorption (25). This study indicates that UA/Cr levels possess significant evaluative significance for osteoporosis in the elderly and surpass the predicted efficacy of SUA levels alone for this condition. In comparison to women, UA/Cr levels exhibit a greater predictive capacity for OP in men. The disparity in the connection between UA/Cr levels and OP can be ascribed to the differing etiologies of OP in men and women (60–62). Elderly women are subjected to a greater number of factors affecting bone mineral density, such as estrogen reduction (63). Further enormous scale prospective researches are required to elucidate the disparities in the relationship between UA/Cr levels and the occurrence of OP in senior individuals across various genders and ethnicities. The UA/Cr index is a straightforward and readily accessible serological diagnostic that can serve as an adjunct in identifying the incidence of OP in the elderly, providing valuable guidance for clinical practice.

## 5 Conclusion

This study utilized a nationally representative sample from NHANES data, ensuring a high level of uniformity and dependability, hence rendering the findings very pertinent to the whole U.S. population. The substantial sample size enabled this study to conduct subgroup analyses to identify disparities among genders and races. Limited research exists regarding the correlation between UA/Cr and OP in the elderly in the U.S., as evidenced by the analysis of NHANES data. UA/Cr can more accurately and sensitively represent the correlation between SUA and OP.

Nevertheless, the study has certain drawbacks. The cross-sectional study restricts the capacity to draw a causal link between UA/Cr and OP in older individuals. Consequently, additional fundamental mechanistic investigations and cohort studies are required to elucidate the precise mechanism of the connection. Additionally, owing to insufficient data and other factors, this study exclusively employed femoral BMD to ascertain the presence of osteoporosis in participants, neglecting BMD measurements in other regions, hence yielding inadequate findings. Finally, there is the potential of bias due to other possible confounding factors that have not been controlled in the study.

In summary, among the aged population in the United States, the UA/Cr ratio exhibits a negative association with the incidence of OP and serves as an independent preventive factor for OP; however, this correlation is limited to Non-Hispanic Whites and is more pronounced in older men. The prognostic value of UA/Cr levels for OP surpasses that of plain SUA. Consequently, in clinical practice, the UA/Cr ratio in the aged population can be routinely assessed to monitor for the onset of OP in patients with markedly diminished UA/Cr levels. Future prospective studies are required to validate the efficacy of UA/Cr in predicting osteoporosis in the elderly.

## Data Availability

Publicly available datasets were analyzed in this study. This data can be found here: https://www.cdc.gov/nchs/nhanes/index.htm.

## Footnotes

1
http://www.Rproject.org
